# Carbonic anhydrase inhibitors (CAIs) decrease cardiac amyloid β pathology, mitigating neuro‐signaling maladaptive remodeling and improving cardiac function in Tg2676‐ AD mouse model

**DOI:** 10.1002/alz.087670

**Published:** 2025-01-03

**Authors:** Andrea Elia, Rebecca M Parodi‐Rullan, Rafael Vazquez‐Torres, Ashley M Carey, Silvia Fossati

**Affiliations:** ^1^ Lewis Katz School of Medicine, Temple University, Philadelphia, PA USA; ^2^ Alzheimer’s Center Lewis Katz School of Medicine, Temple University, Philadelphia, PA USA; ^3^ Alzheimer’s Center at Lewis Katz School of Medicine, Temple University, Philadelphia, PA USA

## Abstract

**Background:**

FDA‐approved carbonic anhydrase inhibitors (CAIs) have been shown to attenuate Aβ pathology, neurodegeneration, and cerebrovascular dysfunction in models of Alzheimer’s disease (AD) and cerebral amyloid angiopathy (CAA), suggesting a key role for CAs as a novel and previously unexplored target for AD therapy. Amyloid β accumulation severely impairs the cerebral neuro‐signaling pathway with a progressive loss in neurotrophic factors (NTFs, i.e., NGF and BDNF), potentially also affecting the cardiac nervous system and culminating in lethal heart dysfunction. Yet, no studies have proposed the use of CAIs for treating and preventing the systemic and cardiac effects of amyloidosis and AD.

**Methods:**

Here, we explored for the first time, the protective effects of a chronic CAIs therapeutic regimen against cardiac amyloid pathology, neuronal fibers’ loss, and heart neurotrophic dysregulation in Tg2576 mice, a widely used model of AD and cerebral amyloidosis.

**Results:**

Cardiac fibrosis enhancement along with progressive Aβ deposition in the heart seriously affected the cardiac neuro‐signaling pathway culminating in a robust decline of BDNF expression associated with myocardial adrenergic nerve fibers and regenerated nerve endings impoverishment (stained with TH and GAP‐43 respectively) in 12‐month‐old Tg2576 mice compared to age‐matched WT littermates. These degenerative mechanisms severely compromised heart function, evidenced by a decline of both ejection fraction and fraction shortening percentage in Tg2576 animals. Accordingly, we observed that human Aβ40 or Aβ42 oligomers’ treatment drastically reduced BDNF and GAP‐43 expression in human neuronal cells and human cardiomyocytes. Notably, long‐term CAIs treatment in Tg2576 mice reduced Aβ accumulation in the heart tissue and reverted cardiac neuro‐signaling pathway adverse remodeling, thus ameliorating heart function, as evidenced through echocardiographic analysis. These *in vivo* results were validated by *in vitro* experiments both in neuronal and cardiac cells.

**Conclusion:**

These findings suggest a new beneficial role of CAIs in preventing the Aβ‐induced cardiovascular remodeling and neuro‐signaling deregulation for the first time in the Tg2576 model of AD. Taken together with the positive effects of CAIs on cerebrovascular function and cognition, this study paves the way for future clinical trials aimed at repurposing FDA‐approved CAIs to prevent and correct both central and peripheral pathologies induced by AD and amyloidosis.

